# Revealing the nuances of ‘Grey Digital Divide’ in Hong Kong: A latent profile analysis

**DOI:** 10.1371/journal.pone.0326413

**Published:** 2025-07-09

**Authors:** Bobo Hi-Po Lau, Eric Ngai-Yin Shum, Alex Pak-Ki Kwok, Ben Chi-Pun Liu, Alex Chi-Keung Chan, Rick Yiu-Cho Kwan, Steve Fu-Fai Fong, Gigi Lam, Chung-Kin Tsang, Daniel Dick-Man Leung, Johnson Chun-Sing Cheung, Jason Tak-Sang Chow, Paulina Pui-Yun Wong, Stuart Gietel-Basten

**Affiliations:** 1 Department of Counselling & Psychology, Hong Kong Shue Yan University, Hong KongChina; 2 Faculty of Social Science, The Chinese University of Hong Kong, Hong KongChina; 3 Department of Social Work, Hong Kong Shue Yan University, Hong KongChina; 4 School of Arts and Humanities, Tung Wah College, Hong KongChina; 5 School of Nursing, Tung Wah College, Hong KongChina; 6 Department of Sociology, Hong Kong Shue Yan University, Hong KongChina; 7 Department of Social Work & Social Administration, The University of Hong Kong, Hong KongChina; 8 Science Unit, Lingnan University, Hong KongChina; 9 Division of Social Science, Hong Kong University of Science & Technology, Hong KongChina; Michigan State University, UNITED STATES OF AMERICA

## Abstract

The ‘grey digital divide’ deprives older adults’ equitable access to information and support, and thereby, their well-being. Policies including subsidies for internet access and devices, digital literacy classes, and telehealth support attempted to close the divide. Yet, it remains doubtful whether the discrepancy could be narrowed, or simply transformed. The mandatory COVID track-and-trace policy, the government’s decade-long digital inclusivity initiatives and the city’s high degree of digitization makes Hong Kong an exemplar for exploring the post-pandemic digital divide. Utilizing a person-centered approach, this study elaborated the intergenerational differences in digital engagement with a random sample of 870 younger (aged 18–54 years) and older (aged 55 years or above) adults (52.1% female) via phone interviews. With 16 indicators of digital motivation, access, digital skills, and usage, latent profile analysis (LPA) yielded three profiles – Proficient, Intermediate, and Novice, with disparate patterns between the younger (90.2%, 8.8%, 0.9%) and the older (59.2%, 35.5%, 5.2%) groups, demonstrating a clear intergenerational divide. Socio-economic status influenced profile membership regardless of age, and that profile membership relates to the frequencies of various social contacts except with family/relatives. Our findings demonstrate how typology defines the needs and assists formulation of segmented interventions toward digital inclusivity. (200 words).

## Introduction

The COVID pandemic accelerated digital device ownership and online activities among older adults as a result of the expansion of online services and digital pandemic policies. A Pew Research Report found an increase in smartphone ownership between 2015 and 2022 in the UK (44% to 73%), Canada (46% to 72%), as well as European countries (e.g., Poland: 13% to 55%, Spain: 51% to 73%, France: 22% to 74%) [1]. In Asia, Singapore experienced a notable rise in smartphone ownership among seniors aged 60 or above, from 74% in 2017 to 89% in 2022 [2]. Seoul reported a smartphone penetration rate of 60.7% among seniors aged 60 and above [3], while 84.0% of Japanese individuals aged 70 or above were using smartphones in 2024 [4]. Hong Kong is also witnessing an increase in older adults’ digital engagement like the global trend alongside its fast-aging population. However, the combination of the pandemic experience of Hong Kong citizens, the compactness of the city, and the government’s decade-long digital support policies generated a unique socio-cultural-technological landscape for elaborating the post-pandemic intergenerational digital divide.

Digital inclusivity refers to the efforts and practices aimed at ensuring equal access and use of digital technologies for all people, regardless of their age, gender, socio-economic background, or abilities [5]. Hong Kong ranks 7th on the recent IMD Digital Competitiveness Index, indicating a relatively high degree of digitization compared to other economies [6]. However, whether such a high degree of digitization translates into digital inclusion remains doubtful. In 2021–2022, the Hong Kong government stipulated a compulsory digital vaccine passport and track-and-trace scheme based on a smart device application (app), known as the LeaveHomeSafe, to control the pandemic. Citizens were required to complete vaccination requirements and check-in with the QR code function of the app through a smart device (i.e., smart phone or tablet) before entering everyday venues, such as restaurants, clinics, schools, markets, and malls. As these venues are vital for the daily functions of most community-dwelling citizens (e.g., food and grocery shopping, socializing, social and healthcare services), this policy, unlike voluntary digital adoption, potentially converted smartphones or smart devices into an essential tool for continuing one’s pre-policy lifestyles and routines. Following this policy, an increase in smartphone ownership, especially among the older adults, was witnessed, from 68.1% in 2020 to 90.7% in 2022, in spite of the hiccups with adoption and usage especially during the first months of its compulsory implementation [7]. Although other Asian economies, including the mainland China, Taiwan, Singapore, and Korea, also implemented robust digital track-and-trace applications to control the pandemic, the compactness of Hong Kong as a city means older adults could be physically close to support for digital access under such rapid digitization, say, through their families living in vicinity or social services in the neighborhood. Furthermore, the Hong Kong government has long implemented a variety of initiatives to enhance digital inclusivity. For instance, the “Smart Silver” Information and Communications Technology (ICT) Outreach Programme for Elders has been implemented since 2014 by the now Digital Policy Office to commission non-profit-making elderly service organizations across the territory to provide digital learning and support programs to older citizens [8]. Despite sharing some socio-cultural-technological characteristics with its regional counterparts that fostered digital engagement of older adults, such as a digital inclusion policy and digital pandemic policy, Hong Kong’s unique mix of demographic (e.g., an aging population), technological (e.g., the high penetration of ICT infrastructure) and social (e.g., digital inclusion and pandemic control policy) factors might have enabled it to be a regional leader in terms of smartphone penetration among older adults, an index that reflects the level of personal, quotidian digital engagement. While these characteristics appeared to favor the city for a narrowing ‘grey digital divide’, that is, the discrepancy between older and younger adults on the access and use of digital technologies [9], age-related gaps are still obvious on more nuanced indicators, such as online monetary transactions, usage of e-government, etc, as per recent studies [10–12].

Digital divide can be reflected on various ‘levels’ from the motivation to access digital media, to skills for navigating the internet and using the device, and to the breadth and depth of usage [9]. Existing studies seldom used a person-centered approach to model how digital divide levels covary to distinguish different pockets of populations. However, such information is particularly useful for finetuning digital policies and services – for instance, some focusing on digital skills and others on digital access – for different targets. Hence, this study typologized digital engagement at various levels of the digital divide (motivation, access, digital skills, and usage) and contrasted their factors and consequences for younger and older adults.

### Resources and appropriation theory of digital divide

According to the Resources and Appropriation Theory (RAT) [9], personal and positional categorical inequalities, such as those due to gender, age, or socio-economic status, result in differential access to resources (e.g., knowledge, support, information, finances), thereby generating the digital divide. Digital divide may operate on successive levels of appropriation of digital technologies, including, the first level, the motivation to use technology (i.e., motivation level); the second level, access to the infrastructure (e.g., the broadband network) and the device (e.g., a smart phone); the third level, digital skills; and the fourth level, usage, operationalized by the duration, frequency, and breadth of using digital technologies. In the increasingly digitalized world, being on the disadvantaged ‘side’ of the digital divide may limit a person’s participation in society, say finding a job, maintaining contacts with close and distant acquaintances, realizing citizenship rights, or even leading a mobile lifestyle (‘digital nomads’). Restrictions in participation in society may reproduce categorical inequalities and differential access to resources. Such a feedback loop explains why the digital divide is consequential. This theory also echoes with International Telecommunication Union (ITU) definition of digital inclusivity [5], covering multiple dimensions of equity from access (distributive), usage (functional) to the ability to capitalize ICT for enhancement of well-being (participatory). The first decades of research on digital divide centered on the access and use of computers and the internet [13,14]. Considering the popularity of smart devices which afford mobility and therefore enhance the breadth and depth of usage, as well as their predominant role in daily lives and pandemic control during COVID, this study focused on the appropriation of smart devices, such as smart phones and tablets, for online activities.

### The grey digital divide

Feeling too old for technologies, fear of new technology, lack of support, cost concerns, worries about cybersecurity as well as problems with physical aging such as declines in sensation, memory, and dexterity often hinder digital engagement in older adults [15,16], and these obstacles remain even after the pandemic related digital transformation [17]. In the European Union, in 2020 while internet access in youths has capped at almost 100%, the percentages averaged at 61% for people aged 65–74 and ranged from 28% in Croatia to 94% in Denmark [18]. Although the access gap due to technological infrastructure (e.g., broadband coverage) may be extinguished with state investment, the access divide may shift to be one concerning the ownership of digital devices, which may significantly affect the efficiency and effectiveness of one’s digital engagement. For instance, older adults are more likely to rely on only one device, such as a smartphone or a tablet, which may restrict the immersiveness and creativity of their digital experience, compared to having a combination of a smartphone, a desktop/ laptop computer, and/or a tablet. Such material access gap is associated with lower digital skills and usage diversity and frequency, even in the Netherlands where the internet penetration rate reached 98% [19]. The skill gap and the usage gap are also often reciprocal [20], such that without a multi-tier support network for an age-friendly digital environment, older adults often feel unwelcomed by the youth-oriented digital culture, leading to a low motivation for learning and usage. Likewise, Lu [21] found that despite the high pervasiveness of digital technologies in Singaporeans’ everyday life, older adults see themselves as ‘less worthy’ digital learners compared to their younger counterparts, as figuratively expressed on the title of her paper ‘We don’t need these fancy things’.

In Hong Kong, the Thematic Household Survey Report No. 77 from the Census & Statistics Department [10] found a marked reduction in weekly internet usage hours (age 45–54 years: 33.3 hours; 55–64 years: 27.2 hours, and 65 years or above: 18.0 hours), online purchase (age 45–54 years: 55.8%; 55–64 years: 31.5%, and 65 years or above: 10.7%), and searching on the web (age 45–54 years: 91.6%; 55–64 years: 84.9%, and 65 years or above: 59.8%) from middle-aged to older groups. For smartphone ownership, the drop primarily occurred at 65 years of age, with 90.7% of people aged 65 years or above having a smartphone while the percentages stood at 99.7% or more for adults aged 55–64 years or younger in 2022. However, the ownership of other digital devices, such as a wearable device (e.g., a smartwatch), remained at just 21.3% among older adults [22]. The awareness of the government’s online services (i.e., GovHK) is also markedly different by age groups (age 45–54 years: 78.2% aware of the service; 55–64 years: 70.5%, and 65 years or above: 39.6%), so as the usage of online government services (age 45–54 years: 96.2%; 55–64 years: 93.8%, and 65 years or above: 68.0%) [10]. Several studies highlighted the difficulties faced by local older adults compared to their younger counterparts when accessing, learning, and using digital technologies. Cheung, Chau, Woo, and Lai [11] reported that despite seeing the benefits of technology to their daily lives, older adults worry about age-related functional decline and keeping pace with rapid digitization, especially during the pandemic. Another qualitative study with middle-aged and older adult students from a diploma course in a local higher institution revealed a high level of confidence in future technology use, but also the necessity of accessible technical and social support for continuous usage [12]. Yang et al [23] also documented an increase in using apps for instrumental purposes (e.g., mobile payment), as compared to recreational, informational, and one-to-many communication purposes among Hong Kong older migrants in the highly digitized Shenzhen (a neighboring city in the Greater Bay Area), citing frequent border crossing as the incentive for increased app usage. Thus, older adults seem to agree that digital technologies are indispensable and instrumental to their daily lives. Yet, access to digital devices, digital skills, and the extent of usage remained divided by age groups, disfavoring older adults.

### Revealing the diversity beneath the grey digital divide with a typology

Older adults are a heterogeneous group, so should how its members engage with the digital world. Thus, people on the disadvantaged side of the digital divide are not homogenous; instead, finer divides exist within an age cohort by other positional categories [24–26]. Besides, some indicators could be more sensitive to the antecedents of digital divide than the others. An early study on the age differences of attitudes to the internet by Loges & Jung [14] found higher age was related to lower access and usage variety of the internet, but not perceived centrality of the internet to users’ life. Using American and Dutch data from 1980s and 1990s, Van Dijk & Hacker [27] contemplated that the discrepancies related to hardware would soon be closed, whereas the skills and usage gaps would expand with the increasingly information- and network-rich society. According to RAT [9], fulfillment of lower-level divides forms the foundation for fulfilling higher-level divides. For instance, having a device on hand (i.e., material access) is the prerequisite for literacy and sustained usage, and eventually ripping the well-being benefits of digital technologies. Considering the cumulative nature of divide level fulfillment and the differences in the sensitivity of divide levels to various antecedents, simultaneously considering a combination of indicators across different levels of the digital divide is useful for realistically depicting and comparing where the dispersion is relatively more (or less) severe. When considering a combination of indicators at the same time, a person-centred approach would be more suitable than a variable-centred approach for modelling individual heterogeneity through assuming the sample as making up of distinct populations that possess different patterns of relationships among the concerned variables, especially across different divide levels. A typology approach can organize these individual differences into definable profiles and facilitate the exploration of the factors behind [28,29].

Latent class modeling is a person-centered, inductive approach to classify individuals into a definable number of classes or profiles [30]. This approach has been used in characterizing the diversity in frequencies of a range of online activities, in other words, the usage divide [31–33]. For instance, Wenz & Keusch [34] constructed a typology of smartphone users considering also self-rated skills, besides online activities. Their German sample of smartphone owners were characterized by six latent classes – advanced users, broad non-social media users, broad non-commercial users, social media and information users, basic general users, and camera users, and the classes were distinguished by gender, age, and education. Their study illustrated how latent profile analysis (LPA) simultaneously organizes the diversity at various levels of digital divide [9] into several profiles and facilitates the exploration of the factors behind these observed differences in digital engagement.

### Research objectives and hypotheses

This study elaborated the post-pandemic ‘grey digital divide’ through a typology approach that simultaneously considers all four levels of digital divide of the RAT [9], including motivation, access, digital skills, and usage. It has three objectives. The first was to generate a typology using LPA to organize individual differences in motivation, access, digital skills, and usage of smart devices and online activities in post-pandemic Hong Kong. Since LPA is an inductive methodology, we did not put forward a hypothesis regarding the resultant number, characteristics, and sizes of profiles.

The second objective was to scrutinize the associations of the resultant digital engagement profiles with demographic variables, self-rated health, and preferences of help-seeking for technology problems. In addition, we looked for whether these variables are associated with digital engagement profiles *differently* among younger and older groups, potentially resulting in distinct patterns of digital divides by age cohort. Based on two rationales, we purposively sampled individuals from two age groups: between aged 18 and 54 years and those aged 55 years or above, and tested how they were different on the distribution of the resultant profiles, and their profiles’ associations with demographics, health, and help seeking. First, in Hong Kong most indicators of online activities (e.g., online purchase, web searching for information, usage hours) began to see a marked drop between the age groups of 45–54 years and 55 years or above based on the recent territory-wide, population-representative Thematic Household Survey Report No. 77 of the Census and Statistics Department [10]. Second, local services for the ‘third age’ and chronic illness management often begin at 55 years of age. Thus, on the one hand, we assumed people aged 55 years or above would be eligible for additional social service support for digital engagement through government’s digital inclusivity initiatives commissioned to elderly services. On the other hand, planned analyses using this age for sub-grouping help yield empirical findings relevant to local service development. As RAT views digital divide as resulted from personal and positional categorial inequalities, we expected more advanced profiles characterized by greater motivation, access, digital skills, and usage to be correlated with more advantageous demographic and health attributes. Besides, we explored whether participants belonging to different digital profiles display differential preferences to sources of help – from family, peers, and social services – when encountering problems with digital technologies. Considering public digital inclusivity initiatives were implemented at social services, ‘social services’ was added as an option alongside ‘families’ and ‘peers’.

Numerous studies found that engagement with the digital world buffered the negative impacts of the pandemic, including social isolation, loneliness, and deteriorating mental health, among older adults [35,36]. The RAT postulates that being on the disadvantageous side of the digital divide jeopardizes participation in society [9]. Among various types of participation stipulated by the theory as the outcomes of the digital divide, social contacts with close bonds such as family and friends, as well as with formal support organizations are robust predictors of health among older adults [37,38]. They are also amenable to change by facilitating the use of digital technologies, especially social media and instant messenger apps. Thus, as the third objective, we tested the associations of resultant profiles with the frequencies of informal and formal social contacts, and explored if these associations could be different among younger and older groups. Likewise, we expected more advanced profiles to correlate with more social contacts.

## Methods

### Ethics Statement

This study received ethical approval from the Human Research Ethics Committee (HREC) at Hong Kong Shue Yan University (HREC 22−05 (M19)). Verbal informed consent was obtained from participants over the phone prior to their participation.

### Sampling and procedure

This study included a purposive sample of Hong Kong adults aged 18 or above, who were cognitively sound, Cantonese-speaking, and living in the community, recruited through random digit dialing to households and mobile phone numbers. A total of 910 responses were collected for the study between December 14, 2023, and January 26, 2024, resulting in a response rate of 42%. After excluding partial responses, the data set for the current analysis contained 870 responses with 431 people aged 18–54 and 439 people aged 55 years or above. A comparable sample size was obtained between the younger (18–54 years) and older (55 years or above) group to facilitate the planned analyses. The total sample size satisfied the recommendations by Nylund et al. [39] and Lo, Mendall & Rubin [40]. Data collection was conducted by a local public polling company, and the interview took about 15–20 minutes to complete.

### Instrument

#### Indicators of digital divide.

Indicators of digital divide followed and were grouped under the four levels stipulated by the RAT, including motivation, access, digital skills, and usage.

#### Motivation.

Motivation to use technologies were evaluated by the optimism and insecurity subscales under the Technology Readiness Index 2.0 [41]. Optimism is a positive view about technology with the belief that it can enhance people’s control, flexibility, and efficiency in their daily lives. Insecurity indicates people’s distrust of technology that originates from their skepticism about its ability to perform as expected and its potential harm. The innovativeness and discomfort subscales were not used as they closely resemble digital skills. Each subscale comprises four statements. Participants rated each statement on a 5-point scale, ranging from 1 (strongly disagree) to 5 (strongly agree). The two subscales demonstrated adequate reliability (Insecurity:.68; Optimism:.80).

#### Material access.

For access, we targeted material access rather than physical access to the internet, as local household broadband penetration reached 98% [42] suggesting a rather peaked level. Material access was measured by participants’ ownership of four types of digital devices, including smartphones, tablets, laptop computers, and smartwatches, and ownership of each type was given a score of 1, yielding a total score that ranged from 0 to 4.

#### Digital skills.

Participants were asked to rate their ability in completing specific tasks: (i) finding useful information on the internet; (ii) using messaging or social media applications; (iii) document handling, (iv) e-payment, and (v) resource sharing. Each item was rated on a 3-point scale, from 1 (do not know how to use it), 2 (haven’t tried it but I know how to use it) to 3 (I have used it before). An additional item on how often the participants solve digital technologies-related problems on their own, such as by referring to online resources or the manual, was asked and responded to on a five-point Likert scale running from 0 (never) to 4 (all the time).

#### Usage.

Usage was evaluated by the duration of device usage and the frequency of key online activities. To assess the duration of device usage, participants were asked to provide the average number of hours per day they used smartphones on weekdays and weekends, as well as the usage hours of laptop on weekdays. Participants were also asked to rate their frequencies of using five categories of smart phone applications on a 5-point scale, range from 0 (never) to 4 (always). The categories are (i) instant messaging or social media; (ii) applications related to money transactions; (iii) health-related applications for medical system or personal health records; (iv) applications related to entertainment; (v) applications for daily life information.

Nine experts in healthcare, social work, gerontology and gerontechnology were solicited to provide feedback on whether the fourteen items (except ‘troubleshooting by oneself’ as well as optimism and insecurity subscales of TRI 2.0) were appropriate for measuring the age-related digital divide in Hong Kong prior to data collection. A criterion validity index of 0.95 was reached considering ‘quite relevant’ and ‘highly relevant’ as positive responses [43]. The item ‘troubleshooting by oneself’ was added based on the suggestions from the experts. Considering the study attempted to fill the extant research gaps by investigating nuanced patterns of achievement of indicators at various digital divide levels, we input the 16 indicators separately into LPA rather than grouping them into subscales (Table 1). These indicators have also been featured as important ones for differentiating younger or more proficient users from older or more novice users in our previous qualitative study with 146 local adults aged 55 or above [44].

**Table 1:** Indicators for latent profile analysis.

Digital divide level	Variables	Range
Motivation	(1) Optimism subscale score (2) Insecurity subscale score	1–5 1–5
Material access	(3) Ownership of four types of digital devices	0–4
Digital skills	(4) Troubleshooting by oneself (5) Finding useful information on the internet (6) Using messaging or social media applications (7) Document handling (8) E-payment (9) Resource sharing	0–4 1–3 1–3 1–3 1–3 1–3
Usage	(10) Instant messaging or social media (11) Applications for money transactions (12) Health-related applications for medical system records or personal health records (13) Applications for entertainment (14) Applications for daily life information (15) Average hours of smart phone usage, daily (16) Average hours of laptops/ computers usage, daily	0–4 0–4 0–4 0–4 0–4 0–24 0–24

#### Demographic factors, health & help-seeking.

Physical health of the participants was rated by a single-item subjective health question using a 5-point scale, which ranged from 1 (excellent) to 5 (very bad). Age, gender, education attainment, occupational status, monthly household income, number of co-living family members and number of immediate families living abroad were collected. Quan-Haase et al. [45] identified the accessibility of social and technological support as a significant obstacle hindering digital skills and inclusion for older individuals. Consequently, participants’ co-residence with family members and whether they have family members living abroad that renders online communication an essential means of contacts were explored as factors of digital engagement. Frequency of seeking help from (i) family, (ii) peers, and (iii) social services for problems related to digital technologies were asked. Participants answered on a scale running from 0 (never) to 4 (always).

#### Social contacts.

Participants were asked to rate their frequencies of contacting (i) family/ relatives; (ii) neighbors/ friends/ co-workers; (iii) schools/ hospitals/ social service agencies; (iv) government departments/ religious organizations/ political groups over the past year on a 4-point scale, range from 1 (never) to 5 (always).

### Statistical analysis

LPA is a type of latent class modelling technique that accommodates continuous variables. We performed the LPA using the tidyLPA R package in the snowRMM function in Jamovi 2.3.2 [46]. LPA was selected over methods such as k-means clustering, hierarchical clustering, or decision trees due to its ability to identify underlying latent classes that are not observable in the data. Besides, while cluster analyses are based on distance measures and rely on ‘hard clustering’ where one person can only be placed in one cluster, LPA derives probabilities of a person belonging to different profiles, in other words, a person can belong to more than one profile with different probabilities (i.e., soft clustering). Soft clustering is likely to provide more nuanced and better fit to the data than hard clustering.

Among different model specification methods, we opted for class-invariant unrestricted parameterization, meaning assuming equal variances and covariances, for balancing the requirements of model fit and parsimony. For models specifying the same number of clusters, more complex models (meaning models that require estimation of more parameters, rather than fixing parameters across resultant profiles) may yield a better fit to the data; yet the increase in complexity may compromise parsimony of the solution. Thus, we opted for equaling both variances and covariances rather than allowing free estimation of these parameters (most complex, least parsimonious) or fixing the covariances to zero (least complex, most restrictive). Class-invariant unrestricted parameterization estimated the variances and covariances of the variables for creating the profiles, thereby offering more information for more comprehensive understanding of the characteristics of the profiles and potentially explaining the data better than specifications that fix covariance to zero, too. Compared to allowing variances and covariances to vary freely, equal-variances-and-equal-covariances also provides more parsimony to the solution. Thus, we reckon that class-invariant unrestricted parameterization offers a balance between model fit and model parsimony. Expectation-maximization (EM) algorithm was used for obtaining maximum likelihood estimates for the parameters, but unlike MPlus where a random starting value is used, tidyLPA uses starting values based on hierarchical clustering for interfacing with R package designed for Gaussian mixture models (mclust).

Next, selecting the optimal number of profiles is also often a balance between the parsimony of the resultant solution and the fulfillment of a combination of fit criteria. Most used criteria in LPA included Bayesian information criteria (BIC), sample-adjusted BIC (SABIC), Bootstrap Likelihood Ratio Test (BLRT), and entropy [47,48]. BIC is preferred over Akaike Information Criterion (AIC) as the former tended to outperform the latter with a large sample size [40] and continuous indicators [49]. However, with a relatively large sample size and number of indicators, additional profiles may result in consistently decreasing BIC. Sinha et al. [48] therefore recommended researchers plotting the BIC values against the number of resultant profiles (i.e., elbow plot) to look for the solution that gives a substantial drop in BIC, in other words, a marked increase in model fit. Good fit is also reflected by the BLRT test. A significant value (e.g., *p* < .05) indicates that the more complex solution (k profiles) provides a significantly better fit than the simpler solution (*k*-1 profiles). Although Tein, Coxe, & Cham [50] suggested when k and k-1-classes both returned with significant BLRT results the k-classes is preferred, they too remarked that SABIC could provide comparable statistical power when the sample size is beyond N = 250. Third, entropy, which can range from 0 to 1.0, informs how well the resultant profiles are separated from one another, and a higher entropy signals a set of better-differentiated profile. Lastly, the decision of an optimal solution is aided by the size of each profile and their conceptual and practical implications.

The associations of the resultant digital engagement profiles with demographic variables, health, and help-seeking were examined by Chi-square tests, t-tests, and one-way ANOVAs. We then used multiple regressions to explore the associations of digital engagement profiles with social contacts, controlling demographic variables and their interactions with age. Upon discovery of significant interactions with age, we proceeded to explore the patterns of associations by younger (18–54 years) and older (55 years or above) groups as planned analyses. All analyses were conducted in Jamovi 2.3.2.

## Results

### Sample characteristics

Table 2 describes the demographic characteristics of the sample and by the two target age groups (18–54 years and 55 years or above). Among the 870 responses, 52.1% identified as female. Approximately 47.6% of the sample had a full-time occupation, and 27.8% were retired. For education, 40.1% completed high school or some post-secondary education, while 41.8% obtained a bachelor’s degree or more. The median monthly household income was HK$30,000–39,999, which was comparable to the general population estimate. Nearly half of the participants (47.6%) did not have any immediate relatives living abroad. Besides being more likely to be retired, compared to their younger counterparts (aged 18–54 years), the older age group (aged 55 years or above) tended to have lower education attainment and household income, and were more likely to have family members living abroad and with fewer co-living family members.

**Table 2:** Sample characteristics by age group.

Variables	N (% within column)			*t* / χ^2^	*p*
Gender, N = 870	Total sample (N = 870)	Aged 18–54 (N = 431)	Aged ≥ 55 (N = 439)		
Female	453 (52.1)	225 (52.2)	228 (51.9)	.01^˅^	.937
**Age, N = 870**					
18 - 24	63 (7.2)	63 (14.6)	–	N/A	N/A
25 - 34	127 (14.6)	127 (29.5)	–		
35 - 44	117 (13.4)	117 (27.1)	–		
45 - 54	124 (14.3)	124 (28.8)	–		
55 - 64	212 (24.4)	–	212 (48.3)		
65 or above	227 (26.1)	–	227 (51.7)		
**Education, N = 870**					
Lacked formal education	6 (0.7)	0 (0.0)	6 (1.4)	11.09	<.001
Primary school	54 (6.2)	3 (0.7)	51 (11.7)		
Junior high school	97 (11.2)	29 (6.7)	68 (15.6)		
Senior high school	246 (28.4)	92 (21.4)	154 (35.3)		
Post-secondary (non-bachelor)	101 (11.7)	60 (14.0)	41 (9.4)		
Post-secondary (bachelor)	256 (29.6)	187 (43.5)	69 (15.8)		
Post-secondary (master/ PhD)	106 (12.2)	59 (13.7)	47 (10.8)		
**Occupation status, N = 870**					
Full-time workers	413 (47.6)	288 (67.0)	125 (28.5)	330.64^˅^	<.001
Part-time workers	75 (8.6)	39 (9.1)	36 (8.2)		
Retired	241 (27.8)	8 (1.9)	233 (53.2)		
Homemaker	68 (7.8)	29 (6.7)	39 (8.9)		
Unemployed	27 (3.1)	22 (5.1)	5 (1.1)		
Full-time students	44 (5.1)	44 (10.2)	0 (0.0)		
**Monthly household income, N = 870**					
HK$9,999 or above	96 (11.7)	13 (3.2)	83 (20.0)	8.97	<.001
HK$10,000 - $19,999	94 (11.5)	27 (6.7)	67 (16.1)		
HK$20,000 - $29,999	113 (13.8)	61 (15.1)	52 (12.5)		
HK$30,000 - $39,999	136 (16.6)	64 (15.8)	72 (17.3)		
HK$40,000 - $59,999	147 (17.9)	90 (22.2)	57 (13.7)		
HK$60,000 - $79,999	95 (11.6)	66 (16.3)	29 (7.0)		
HK$80,000 or above	139 (17.0)	84 (20.7)	55 (13.3)		
**Number of immediate relatives residing outside Hong Kong, N = 870**					
None	410 (47.6)	254 (59.3)	156 (35.9)	−6.40	<.001
1 - 2	198 (23.0)	86 (20.1)	112 (25.8)		
3 - 4	97 (11.3)	27 (6.3)	70 (16.1)		
5 or above	157 (18.2)	61 (14.3)	96 (22.2)		
	**Mean (SD)**				
**Number of co-living family members**	2.22 (1.43)	2.50 (1.42)	1.95 (1.39)	5.75	<.001
**Self-rated health** ^×^	2.53 (0.84)	2.58 (0.83)	2.48 (0.84)	1.67	.095

˅ Chi-square test was conducted. ^×^ Measured on a 5-point scale, from 1 (excellent) to 5 (very bad). *t*: Student’s *t*

Table 3 provides the characteristics of digital engagement by the 16 indicators among the whole sample and by the younger and older age groups. The indicators were modestly correlated with one another with Pearson’s *r*s ranging from 0.015 (duration of smart phone use and use of apps for health records) to 0.660 (skill of e-payment and usage of apps for financial transaction) (See Table 1 in S1 Table for the intercorrelations), even when the pair belonged to the same digital divide level, supporting the use of a typology approach. Of note, optimism was positively correlated with all indicators of app usage, range of device ownership, and all literacy indicators, except literacy on social media and average laptop usage. Insecurity was only negatively correlated with troubleshooting by oneself, usage of financial apps and literacy on resource sharing. The younger (aged 18–54 years) and the older (aged 55 year or above) groups were significantly different in all indicators except insecurity (motivation) and frequency of using apps for health purposes (usage). Compared to the younger group, the older group were less optimistic about technology (motivation level), owned a narrower range of devices (access level), perceived lower digital skills, used the devices for fewer hours, and were less frequent users of a variety of apps. Of note, the average weekly hours spent on smartphones was about 26.32 hours (3.76 x 7 days) among the older groups and 37.94 hours (5.42 x 7 days) among the younger groups, which were comparable to the findings of the Thematic Household Survey Report No. 77 from the Census & Statistics Department [10] (i.e., aged 25–34 years: 41.9 hours; 35–44 years: 38.3 hours, 45–54 years: 33.3, 55-64 years: 27.2 hours).

**Table 3:** Characteristics of digital motivation, material access, skill and usage by age group.

Variables	Mean (SD)			*t*	*p*	Mean (SD)		*t*	*p*
	Total sample (N = 870)	Aged 18–54 (N = 431)	Aged ≥ 55 (N = 439)			Aged 55–64 (N = 212)	Aged ≥ 65 (N = 227)		
**Motivation**									
Optimism subscale score^+^	3.96 (0.80)	4.01 (0.72)	3.91 (0.86)	1.99	.047	3.98 (0.76)	3.84 (0.94)	1.75	.081
Insecurity subscale score^+^	3.52 (0.80)	3.51 (0.69)	3.52 (0.89)	−0.23	.819	3.58 (0.77)	3.48 (0.98)	1.19	.233
**Material access**									
Ownership of four types of digital devices ^o^	2.60 (1.03)	2.91 (0.94)	2.30 (1.02)	9.29	<.001	2.46 (1.02)	2.15 (1.00)	3.24	.001
**Digital skills**									
Troubleshooting by oneself ^^^	2.02 (1.38)	2.63 (1.13)	1.42 (0.76)	14.92	<.001	1.81 (1.27)	1.06 (1.12)	6.53	<.001
Finding useful information on the internet ^#^	2.78 (0.60)	2.93 (0.32)	2.64 (0.76)	7.55	<.001	2.81 (0.58)	2.48 (0.86)	4.70	<.001
Using messaging or social media applications ^#^	2.95 (0.86)	2.99 (0.10)	2.91 (0.41)	4.16	<.001	2.95 (0.29)	2.86 (0.49)	2.30	.022
Document handling ^#^	2.48 (0.86)	2.79 (0.58)	2.18 (0.98)	11.41	<.001	2.48 (0.86)	1.89 (0.97)	6.72	<.001
E-payment ^#^	2.57 (0.77)	2.89 (0.40)	2.26 (0.91)	13.23	<.001	2.53 (0.79)	2.01 (0.94)	6.21	<.001
Resource sharing ^#^	2.05 (0.93)	2.57 (0.76)	1.55 (0.79)	19.43	<.001	1.74 (0.87)	1.37 (0.66)	5.10	<.001
**Usage**									
Instant messaging or social media^^^	2.83 (0.90)	3.13 (0.74)	2.54 (0.95)	10.15	<.001	2.76 (0.78)	2.34 (1.04)	4.74	<.001
Applications for money transactions ^^^	1.82 (1.25)	2.31 (1.04)	1.34 (1.26)	12.30	<.001	1.79 (1.22)	0.93 (1.14)	7.70	<.001
Health-related applications for medical system records or personal health records ^^^	1.30 (1.09)	1.27 (1.08)	1.33 (1.10)	−0.74	.462	1.39 (1.09)	1.26 (1.11)	1.21	.226
Applications for entertainment ^^^	1.99 (1.16)	2.30 (1.02)	1.69 (1.21)	8.01	<.001	1.84 (1.18)	1.55 (1.23)	2.47	.014
Applications for daily life information^^^	2.08 (0.98)	2.29 (0.88)	1.88 (1.03)	6.21	<.001	2.09 (0.96)	1.70 (1.06)	4.03	<.001
Average hours of smart phone usage, daily	4.58 (3.41)	5.42 (3.55)	3.76 (3.07)	7.36	<.001	4.26 (3.39)	3.30 (2.65)	3.33	<.001
Average hours of laptop/ computer usage, daily	3.38 (3.81)	4.66 (3.93)	2.11 (3.22)	10.46	<.001	2.92 (3.50)	1.36 (2.74)	5.19	<.001

+ Measured on a 5-point scale, from 1 (strongly disagree) to 5 (strongly agree); ^O^ Material access was assessed by participants’ ownership of four digital devices: smartphones, tablets, laptops, and smartwatches, each scored 1, for a total range of 0–4; ^#^ Measured on a from 1 (do not know how to use it), 2 (haven’t tried it but I know how to use it) to 3 (I have used it before); ^ Measured on a 5-point scale, from 0 (Never) to 4 (Always); *t*: Student’s *t*

For a more nuanced investigation of the digital divide by age within the older group, we conducted independent sample t-tests to explore the differences between people aged 55–64 years and 65 years or above. While optimism and insecurity were comparable, people aged 65 years or above scored lower on material access, all items of digital skills, and most usage items, except using apps for health purposes.

### Latent profiles of digital engagement

We submitted these 16 indicators of the four levels of digital divide (motivation, access, digital skills, and usage) to an LPA to generate a typology of digital engagement for the whole sample. Table 4 provides the results of LPA solutions with successive number of latent profiles. To balance the desirability for a parsimonious solution and the model fit revealed by a combination of statistical criteria, we found the three-profile solution most desirable (Table 4). No solution provided a non-significant BLRT result. Yet, considering the relatively large sample size and number of indicators, the three-profile solution provided the largest drop in BIC and SABIC compared to other solutions indicating the largest increase in model stability as the solution complexity increases. The three-profile solution also possesses the highest entropy indicating a well-differentiated set of latent profiles. Besides, the three resultant profiles are of adequate sizes to be conceptually and practically robust [51].

**Table 4:** Results of latent profile analysis.

Class	LogLik	AIC	AWE	BIC	CAIC	SABIC	Entropy	BLRT *p* value
1	−18001	36306	38514	37031	37183	36548	1	0.010
2	−17131	34600	37055	35407	35576	34870	0.989	0.010
3	−16158	32688	35390	33575	33761	32985	0.993	0.010
4	−15817	32041	34990	33009	33212	32247	0.977	0.010
5	−15745	31930	35127	32979	33199	32281	0.967	0.010
6	−15366	31205	34649	32336	32573	31583	0.954	0.010

LogLik: Extract Log-Likelihood; AIC: Akaike Information Criterion; AWE: Approximate weight of evidence; BIC: Bayesian Information Criterion; CAIC: Consistent Aikake information criterion; SABIC: Sample size-adjusted BIC; BLRT: bootstrapped likelihood ratio test.

Non-significant BLRT value suggests that the simpler model provides a reasonably good fit to the data, and the additional complexity of the more complex model is not warranted. Entropy is a standardized index of model-based classification accuracy, with higher values indicating more precise assignment of individuals to latent profiles. A lower Bayesian Information Criterion (BIC) value indicates a more parsimonious statistical model that achieves a favorable balance between goodness of fit and model complexity.

**Fig 1:**
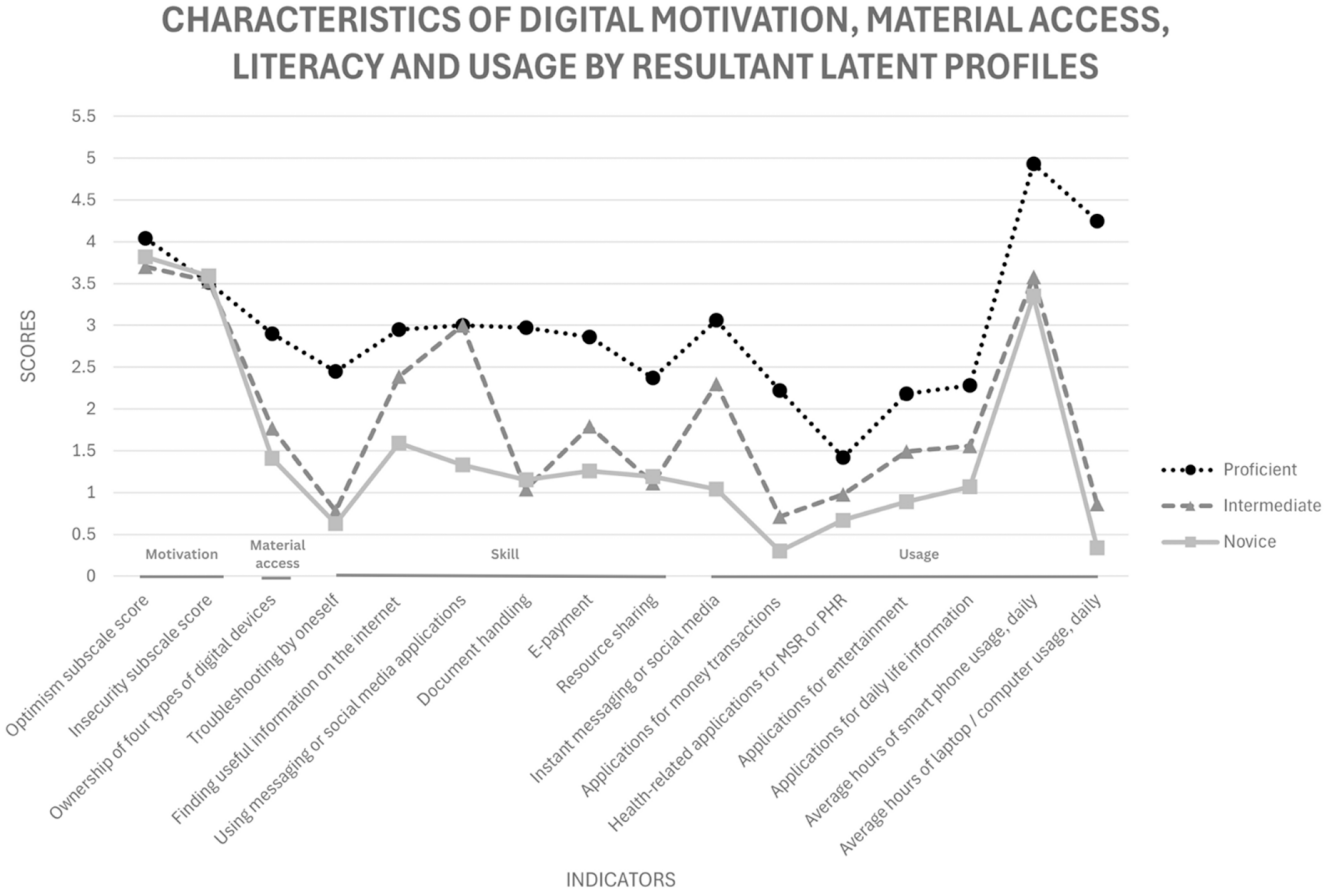
Characteristics of digital motivation, material access, skill and usage by resultant latent profiles. MSR: Medical System Records; PHR: Personal Health Records.

We labelled the three profiles as ‘Proficient’ (n = 649, 74.6%), ‘Intermediate’ (n = 194, 22.3%), and ‘Novice’ (n = 27, 3.1%). We illustrated how the three profiles differed on the 16 indicators (Fig 1). Inter-profile comparisons were conducted by ANOVA with Tukey’s HSD test for post-hoc analysis (Table 5).

**Table 5:** Characteristics of digital motivation, material access, skill and usage by resultant latent profiles.

Variables	Latent profiles			*F*	*p*
	Mean (SD)				
	Proficient (n = 649)	Intermediate (n = 194)	Novice (n = 27)		
**Motivation**					
Optimism subscale score^+^	4.04 (0.70)	3.70 (0.98)^bb^	3.82 (1.00)	14.76	<.001
Insecurity subscale score^+^	3.51 (0.74)	3.53 (0.91)	3.59 (1.14)	0.17	0.845
**Material access**					
Ownership of four types of digital devices^O^	2.90 (0.91)^aa^	1.77 (0.84)^bb^	1.41 (0.64)	146.21	<.001
**Skill**					
Troubleshooting by oneself^^^	2.45 (1.17)^aa^	0.78 (0.95)^bb^	0.63 (1.01)	188.41	<.001
Finding useful information on the internet^#^	2.95 (0.28)^aa^	2.39 (0.90)^bb^	1.59 (0.84)^cc^	164.67	<.001
Using messaging or social media applications^#^	3.00 (0.00)^aa^	3.00 (0.00)	1.33 (0.48)^cc^	5250.58	<.001
Document handling^#^	2.97 (0.18)^aa^	1.04 (0.20)^b^	1.15 (0.46)^cc^	7837.31	<.001
E-payment^#^	2.86 (0.44)^aa^	1.79 (0.92)^bb^	1.26 (0.59)^cc^	321.43	<.001
Resource sharing^#^	2.37 (0.84)^aa^	1.11 (0.37)^bb^	1.19 (0.56)	228.73	<.001
**Usage**					
Instant messaging or social media^^^	3.06 (0.68)^aa^	2.30 (1.02)^bb^	1.04 (1.09)^cc^	143.91	<.001
Applications for money transactions^^^	2.22 (1.05)^aa^	0.71 (1.06)^bb^	0.30 (0.72)	185.11	<.001
Health-related applications for medical system records or personal health records^^^	1.42 (1.07)^aa^	0.98 (1.07)^bb^	0.67 (0.92)	17.68	<.001
Applications for entertainment^^^	2.18 (1.07)^aa^	1.49 (1.26)^bb^	0.89 (1.01)^c^	42.43	<.001
Applications for daily life information^^^	2.28 (0.85)^aa^	1.56 (1.08)^bb^	1.07 (1.11)^c^	63.94	<.001
Average hours of smart phone usage, daily	4.93 (3.38)^a^	3.58 (3.22)^bb^	3.35 (3.99)	13.59	<.001
Average hours of laptop/ computer usage, daily	4.25 (3.84)^aa^	0.86 (2.30)^bb^	0.34 (1.00)	80.80	<.001

^a^Proficient & Novice Post-Hoc Test significant, *p* < .05; ^aa^ *p *< .01.

^b^Proficient & Intermediate Post-Hoc Test significant, *p* < .05; ^bb^ *p *< .01.

^c^Novice & Intermediate Post-Hoc Test significant, *p* < .05; ^cc^ *p *< .01.

+ Measured on a 5-point scale, from 1 (strongly disagree) to 5 (strongly agree); ^O^ Material access was assessed by participants’ ownership of four digital devices: smartphones, tablets, laptops, and smartwatches, each scored 1, for a total range of 0–4; ^#^ Measured on a from 1 (do not know how to use it), 2 (haven’t tried it but I know how to use it) to 3 (I have used it before); ^ Measured on a 5-point scale, from 0 (Never) to 4 (Always);

#### Proficient: Optimistic and cross-domain usage.

Participants in Proficient were the most optimistic about technologies, seeing technologies as good for the livelihood of people and society. They were likely to own two additional digital devices besides their smart phones, were aware of and/or had experience with all digital skills items. They had the highest tendency to trouble-shoot by oneself and were frequently using apps for a breadth of functions, from communication on social media to handling money matters online, except for health purposes which scored below the mid-point on the response scale.

#### Intermediate: Cautious and focus on instant communication.

Participants in Intermediate were, however, the least likely to see technologies as good for oneself and society, as shown by the lowest optimism scores. They owned about two digital devices, including a smartphone. They were good at using social media, like Proficient, but seldom solved technological problems on their own. While most had the experience of searching for information on the web, a majority of them did not attempt document handling and resource sharing, as the averages of these skill items were all below 2 and were the lowest among the three groups. For usage, Intermediate were also using apps for a variety of functions, but with lower frequencies than Proficient. Among them, Intermediate used instant messaging or social media the most, which were the only item with a mean score above the midpoint. However, apps for money transactions and health purposes scored below 1, indicating a low usage frequency.

#### Novice: Minimal exposure.

Participants in Novice ranked middle on optimism. They mostly owned just a smartphone, and their digital skills were the lowest, except for document handling and resource sharing, with which the former was slightly better than Intermediate. E-payment was another skill item that they struggled with, scoring the third lowest among the six skills items. Like Intermediate, they were also unlikely to solve technological problems on their own. Except instant messaging or social media and apps for daily life information (e.g., transportation), Novice had minimal usage with most categories of apps, as reflected by mean scores about 0 (never) to 1 (seldom) on the usage items. Like their counterparts in Intermediate, they were unlikely to use apps for money transactions.

In terms of the duration of device use, Proficient used about 5 hours of smart phone per day, significantly higher than those of Intermediate and Novice which was about 3 hours. However, Proficient were using computers for a much longer period per day (i.e., 4.25 hours) than Intermediate and Novice, which were less than 1 hour.

### Association of digital engagement profiles with demographic, health and help-seeking

The distribution of the three profiles were substantially different between the younger (aged 18–54 years) and the older (aged 55 years or above) age groups, χ^2^(1) = 53.83, *p* < .001. Among the older group, 59.2% (n = 260) were Proficient, 35.5% (n = 156) were Intermediate, and 5.2% (n=23) were Novice. Among the younger group, the corresponding percentages were 90.2% (n = 389), 8.8% (n = 38), and 0.9% (n = 4). Table 6 presents the distribution of these three profiles across the six age groups: 18–24, 25–34, 35–44, 45–54, 55–64, and 65 or above. The proportion of Proficient decreased, while those of Intermediate and Novice increased with increasing age, and obvious changes were noted at 35–44, 55–64, and 65 or above.

**Table 6:** Distribution of the three profiles in each age bracket.

Age Groups (years of age)	Proficient n (%)	Intermediate n (%)	Novice n (%)	Total N (%)
18 - 24	63 (100.0)	0 (0.0)	0 (0.0)	63
25 - 34	124 (97.6)	2 (1.6)	1 (0.8)	127
35 - 44	98 (83.8)	17 (14.5)	2 (1.7)	117
45 - 54	104 (83.9)	19 (15.3)	1 (0.8)	124
55 - 64	159 (75.0)	47 (22.2)	6 (2.8)	212
65 or above	101 (44.5)	109 (48.0)	17 (7.5)	227
Total	649 (74.6)	194 (22.3)	27 (3.1)	870

As per the planned analyses by the younger (aged 18–54 years) and older (aged 55 or above) age groups, Table 7 describes other demographic and health factors that differentiated the three profiles. Among the older group (aged 55 years or above), Proficient were more advantaged in terms of education, employment and income than Intermediate, and then Novice, whereas the proportion of females was the highest in Intermediate. Proficient reported a higher number of co-living family members than Novice and Intermediate. Novice were the most likely to have no family members abroad, while 43.8% of Intermediate had three or more family members abroad. Proficient had better self-ratings of health than Intermediate, and then Novice. All demographics and health factors significantly differentiated the three profiles.

**Table 7:** Sample characteristics by resultant latent profiles.

Variables	Aged 18–54 (N = 431)					Aged ≥ 55 (N = 439)				
	Classes			*F*/ χ^2^	*p*	Classes			*F*/ χ^2^	*p*
	Proficient (N = 389)	Intermediate (N = 38)	Novice (N = 4)			Proficient (N = 260)	Intermediate (N = 156)	Novice (N = 23)		
**Gender, N = 870**	N (% within column)					N (% within column)				
Female	197 (50.6)	27 (71.1)	1 (25.0)	6.89^˅^	.031	122 (46.9)	94 (60.3)	12 (52.2)	6.94^˅^	.031
**Age**	**N = 431**					**N = 439**				
18 - 24	63 (16.2)	0 (0.0)	0 (0.0)	11.56	<.001	–	–	–	23.29	<.001
25 - 34	124 (31.9)	2 (5.3)	1 (25.0)			–	–	–		
35 - 44	98 (25.2)	17 (44.7)	2 (50.0)			–	–	–		
45 - 54	104 (26.7)	19 (50.0)	1 (25.0)			–	–	–		
55 - 64	–	–	–			159 (61.2)	47 (30.1)	6 (26.1)		
65 or above	–	–	–			101 (38.8)	109 (69.9)	17 (73.9)		
**Education**	**N = 430**					**N = 436**				
Lacked formal education	0 (0.0)	0 (0.0)	0 (0.0)	51.86	<.001	1 (0.4)	3 (1.9)	2 (8.7)	105.89	<.001
Primary school	1 (0.3)	2 (5.3)	0 (0.0)			5 (1.9)	39 (25.0)	7 (30.4)		
Junior high school	13 (3.4)	16 (42.1)	0 (0.0)			13 (5.1)	48 (30.8)	7 (30.4)		
Senior high school	73 (18.8)	17 (44.7)	2 (50.0)			100 (38.9)	47 (30.1)	7 (30.4)		
Post-secondary (non-bachelor)	60 (15.5)	0 (0.0)	0 (0.0)			30 (11.7)	11 (7.1)	0 (0.0)		
Post-secondary (bachelor)	183 (47.2)	2 (5.3)	2 (50.0)			63 (24.5)	6 (3.8)	0 (0.0)		
Post-secondary (master/ PhD)	58 (14.9)	1 (2.6)	0 (0.0)			45 (17.5)	2 (1.3)	0 (0.0)		
**Occupation status**	**N = 430**					**N = 438**				
Full-time workers	270 (69.6)	16 (42.1)	2 (50.0)	95.19^˅^	<.001	101 (39.0)	22 (14.1)	2 (8.7)	51.89 ^˅^	<.001
Part-time workers	32 (8.2)	7 (18.4)	0 (0.0)			22 (8.5)	14 (9.0)	0 (0.0)		
Retired	5 (1.3)	1 (2.6)	2 (50.0)			123 (47.5)	95 (60.9)	15 (65.2)		
Homemaker	18 (4.6)	11 (28.9)	0 (0.0)			10 (3.9)	24 (15.4)	5 (21.7)		
Unemployed	19 (4.9)	3 (7.9)	0 (0.0)			3 (1.2)	1 (0.6)	1 (4.3)		
Full-time students	44 (11.3)	0 (0.0)	0 (0.0)			0 (0.0)	0 (0.0)	0 (0.0)		
**Monthly household income**	**N = 405**					**N = 415**				
HK$9,999 or below	9 (2.5)	4 (11.4)	0 (0.0)	20.35	<.001	28 (11.4)	48 (32.0)	7 (35.0)	30.85	<.001
HK$10,000 - $19,999	21 (5.7)	6 (17.1)	0 (0.0)			30 (12.2)	30 (20.0)	7 (35.0)		
HK$20,000 - $29,999	47 (12.8)	13 (37.1)	1 (33.3)			33 (13.5)	16 (10.7)	3 (15.0)		
HK$30,000 - $39,999	57 (15.5)	7 (20.0)	0 (0.0)			43 (17.6)	26 (17.3)	3 (15.0)		
HK$40,000 - $59,999	84 (22.9)	4 (11.4)	2 (66.7)			39 (15.9)	18 (12.0)	0 (0.0)		
HK$60,000 - $79,999	66 (18.0)	0 (0.0)	0 (0.0)			25 (10.2)	4 (2.7)	0 (0.0)		
HK$80,000 or above	83 (22.6)	1 (2.9)	0 (0.0)			47 (19.2)	8 (5.3)	0 (0.0)		
**Number of immediate relatives residing outside Hong Kong**	**N = 428**					**N = 434**				
None	239 (61.8)	13 (35.1)	2 (50.0)	10.60	<.001	94 (36.6)	51 (32.9)	11 (50.0)	2.08	.127
1 - 2	79 (20.4)	6 (16.2)	1 (25.0)			72 (28.0)	36 (23.2)	4 (18.2)		
3 - 4	23 (5.9)	4 (10.8)	0 (0.0)			42 (16.3)	25 (16.1)	3 (13.6)		
5 or above	46 (11.9)	14 (37.8)	1 (25.0)			49 (19.1)	43 (27.7)	4 (18.2)		
**Number of co-living family members**	2.46 (1.40)	2.87 (1.49)	2.75 (2.63)	1.50	.224	2.10 (1.30)	1.79 (1.50)	1.39 (1.31)	4.44	.012
**Self-rated health** ^**#**^	2.56 (0.83)	2.76 (0.82)	2.50 (1.29)	1.07	.343	2.40 (0.75)	2.57 (0.93)	2.78 (1.04)	3.61	.028
**When you encounter problems with information technology products, how often do you seek assistance from the following sources?**										
**Family**^	0.61 (0.93)	1.76 (1.44)^b^	1.25 (1.50)	24.27	<.001	1.45 (1.14)	1.76 (1.14)^b^	1.30 (1.02)	4.22	.015
**Peers**^	1.21 (1.06)	0.97 (0.94)	0.50 (0.58)	1.71	.182	1.17 (0.95)	1.11 (1.03)	0.78 (1.00)	1.72	.181
**Social services**^	0.09 (0.34)	0.29 (0.77)^b^	0.00 (0.00)	4.67	.010	0.15 (0.49)	0.36 (0.80)^b^	0.17 (0.65)	5.75	.003

˅ Chi-square test was conducted. ^#^ Measured on a 5-point scale, from 1 (excellent) to 5 (very bad); ^ Measured on a 5-point scale, from 0 (Never) to 4 (Always);

^a^Proficient & Novice Post-Hoc Test significant, *p* < .05; ^aa^ *p *< .01;

^b^Proficient & Intermediate Post-Hoc Test significant, *p* < .05; ^bb^ *p *< .01;

^c^Novice & Intermediate Post-Hoc Test significant, *p* < .05; ^cc^ *p *< .01

For the younger group (aged 18–54 years), again, Proficient was more likely to be younger, educated, and more likely to be working full-time rather than being retired, and were having a higher income than Intermediate and Novice. Intermediate had more females than Novice and Proficient. Also, Intermediate were more likely to have three or more immediate family members living abroad, but the three groups were not significantly different on the number of co-residing family members and self-rated health.

Our findings on help-seeking preferences indicate that individuals with an Intermediate profile, in both younger and older age groups, sought help from family and social services more frequently than those with other profiles. However, the overall frequencies were low, ranging from 0 (never) to 1 (rarely). Post-hoc tests results of ANOVA revealed that participants with an Intermediate profile requested assistance from family (*p* < .05) and social services (*p* < .05) significantly more often than those with a Proficient profile. In both age groups, participants with a Novice profile did not show significant differences in help-seeking behavior from family (*p* > .05) and social services (*p* > .05) compared to Proficient participants. Similarly, no notable differences were found when comparing Novice participants to Intermediate participants across both age groups (*p* > .05). Additionally, no significant differences were observed among participants from the three profiles regarding seeking assistance from peers.

### Association of digital engagement profiles with social contacts

Table 8 illustrates the frequencies of different types of social contacts by the three digital engagement profiles. Generally speaking, more advanced digital engagement profiles were related to higher frequencies of different types of social contacts. To elaborate the association between digital engagement profiles and social contacts, a multiple linear regression analysis was performed for each type of social contact as an outcome variable, utilizing the total sample controlling demographic variables, as well as their interaction with age (see Table 9). Digital engagement profiles continued to emerge as significant predictors in most models, with Novice reporting a lower frequency than Proficient for contacts with neighbors/friends/co-workers and schools/ hospitals/ social service agencies, and Intermediate having fewer contacts with government departments/ religious organizations/ political groups than Proficient. However, digital engagement profile was not a significant predictor for contacts with family/relatives. Furthermore, education, occupational status, and number of immediate relatives residing outside Hong Kong were also related to the frequencies of different types of social contacts.

**Table 8:** Frequencies of social contacts by digital engagement profiles and age groups.

	Total Sample			Younger (Aged 18–54 years)			Older (Aged 55 or above)		
**Digital engagement profiles**	**Proficient (N = 649)**	**Intermediate** **(N = 194)**	**Novice** **(N = 27)**	**Proficient** **(N = 389)**	**Intermediate** **(N = 38)**	**Novice** **(N = 4)**	**Proficient** **(N = 260)**	**Intermediate** **(N = 156)**	**Novice** **(N = 23)**
**Contact with family/relatives^**									
**M(SD)**	3.67 (0.65)	3.45 (0.83)	3.33 (0.92)	3.68 (0.65)	3.34 (0.94)	3.25 (0.96)	3.65 (0.66)	3.47 (0.81)	3.35 (0.94)
**Contact with neighbors/friends/co-workers^**									
**M(SD)**	3.46 (0.77)	3.17 (0.94)	2.67 (1.78)	3.51 (0.76)	3.16 (0.89)	2.00 (0.82)	3.40 (0.79)	3.17 (0.95)	1.85 (0.86)
**Contact with schools/ hospitals/ social service agencies^**									
**M(SD)**	2.48 (1.03)	2.29 (1.05)	1.85 (0.86)	2.38 (1.07)	2.13 (1.07)	1.75 (0.96)	2.54 (0.98)	2.33 (1.04)	1.87 (0.87)
**Contact with government departments/ religious organizations/ political groups^**									
**M(SD)**	2.01 (1.07)	1.71 (0.97)	1.44 (0.75)	1.93 (1.06)	1.47 (0.73)	1.50 (1.00)	2.14 (1.08)	1.76 (1.02)	1.44 (0.73)

^ Measured on a 5-point scale, from 0 (Never) to 4 (Always)

**Table 9:** Regression models for predicting social contact with interaction with age.

Outcomes variables	Contact with family /relatives	Contact with neighbors/friends/co-workers	Contact with schools/ hospitals/ social service agencies	Contact with government departments/ religious organizations/ political groups
Gender: Female (Male as reference)	.185	.255	.080	.065
Age	.093	-.007	.123	.096
Education	.125*	.073*	.124*	.121
Occupational status: Full-time/ Part-time workers (Others as reference)	.000	-.069*	.060	.010
Monthly household income	.117	.144	.015	.072
Number of immediate relatives residing outside Hong Kong	.010	.003*	.028	.013
Number of co-living family members(s)	.138	.007	.033	.005
Self-rated health (Higher scores, poorer health)	−.110	-.173	.082	−.001
Age* Gender: Female (Male as reference)	−.034	.051	−.003	.090
Age* Education level	-.102*	-.090*	-.066	.092*
Age* Occupational status: Full-time/ Part-time workers (Others as reference)	-.011	.057	-.042	.024
Age* Monthly household income	.039	.075	−.011	−.045
Age* Number of immediate relative residing outside Hong Kong	.083*	.081*	.026	.015
Age* Number of co-living family members	.009	.054	.007	.017
Age* Self-rated health	-.059	−.013	.018	.022
Profile: Novices (Proficient as reference)	−.105	−.550*	−.526*	−.388
Profile: Intermediate (Proficient as reference)	−.073	−.109	−.160	−.220*
*F* (DF1, DF2)	5.76 (17, 797)	7.73 (17, 798)	1.80 (17, 798)	3.12 (17, 798)
*p*	<.001	<.001	.024	<.001

**β**: Standardized coefficient.

*p < .05;**p < .01.

The interaction between age and education was a significant predictor in most models, except for the one involving schools/ hospitals/ social service agencies. Higher age appeared to suppress the positive association between education and contacts for family/relatives and neighbors/friends/co-workers, but strengthens the positive association with contacts with government departments/ religious organizations/ political groups. The significant interaction between age and the number of immediate relatives residing outside Hong Kong, suggested that age reinforces the positive association between the number of immediate relatives residing outside Hong Kong and contacts with family/relatives and with neighbors/friends/co-workers. Other interaction effects were not significant.

Since significant interactions with age were found, we proceeded to the planned analyses by the two age groups (aged 18–54 years and aged 55 years or above). As the occupational make-up of the younger and the older groups was vastly different (e.g., more retirees in the older group, and full-time students in the younger group), we separated occupational status into finer categories in the subsequent analyses. Table 10 provides the results of the multiple regressions that explored the association of profiles with social contacts controlling demographic variables and by the two age groups. For the older group, profile was a significant predictor only with schools/ hospitals/ social service agencies, where Novice has fewer contacts than Proficient. Profile was not a significant predictor in other models. Other significant predictors of different types of social contacts included age, number of co-living relatives, monthly household income, self-rated health, and gender. For the younger group, digital engagement profile was a significant predictor for contacts with neighbors/ friends/ coworkers, and the contrast showed Novice having fewer contacts than Proficient. Digital engagement profile was also a significant predictor of contacts with departments/ religious organizations/ political groups, with Intermediate having fewer contacts than Proficient. Yet, the overall model was not significant for the contact with departments/ religious organizations/ political groups. Other profile-contact associations were non-significant. Besides digital engagement profile, gender, education, number of co-residing family members, self-rated health and occupational status were significantly related to various types of social contacts.

**Table 10:** Regression models for predicting social contact by age groups.

Outcomes variables	Contact with family /relatives		Contact with neighbors/friends/co-workers		Contact with schools/ hospitals/ social service agencies		Contact with government departments/ religious organizations/ political groups	
	**Younger** **(Aged 18–54 years)**	**Older (Aged 55 years or above)**	**Younger** **(Aged 18–54 years)**	**Older (Aged 55 years or above)**	**Younger** **(Aged 18–54 years)**	**Older (Aged 55 years or above)**	**Younger** **(Aged 18–54 years)**	**Older (Aged 55 years or above)**
Gender: Female (Male as reference)	.208*	.179	.158	.326*	.054	.075	−.027	.175
Age	−.061	.141*	−.043	.085	.104	.062	.061	.076
Education level	.184*	.035	.067	.043	.154*	.046	−.020	.256**
Occupational status: Part-time workers (Full-time workers as reference)	.063	−.052	−.231	.031	.051	−.057	−.137	−.095
Occupational status: Retired (Full-time workers as reference)	−.159	−.158	−.009	−.087	.297	.065	.401	−.014
Occupational status: Homemaker (Full-time workers as reference)	.382	−.313	−.038	−.036	.226	.012	.028	−.078
Occupational status: Unemployed (Full-time workers as reference)	.233	−.879	−.569*	−.307	−.124	.002	−.024	−.431
Occupational status: Full-time students (Full-time workers as reference)	−.098	N/A	−.373	N/A	.433*	N/A	−.179	N/A
Monthly household income	.101	.137*	.105	.172*	.060	−.035	.128*	−.001
Number of immediate relatives residing outside Hong Kong	−.063	.066	−.052	.067	.016	.046	.028	.005
Number of co-living family members	.104*	.171**	−.043	.061	.004	.052	−.012	.038
Self-rated health (Higher scores, poorer health)	−.043	−.168**	−.174**	−.168**	.053	.115*	−.021	.025
Profile: Novices (Proficient as reference)	−.205	−.015	− 1.508*	−.415	−.419	−.651*	−.588	−.338
Profile: Intermediate (Proficient as reference)	−.138	−.079	−.141	−.100	−.048	−.225	−.484*	−.120
*F* (DF1, DF2)	3.46 (14,387)	5.19 (13,399)	4.47 (14, 387)	4.69 (13,400)	1.44 (14, 387)	1.39 (13, 400)	1.56 (14, 387)	2.98 (13, 400)
*p*	<.001	<.001	<.001	<.001	.133	.162	.088	<.001

β: Standardized coefficient.

*p < .05;**p < .01.

## Discussion

This study attempted to elaborate the post-pandemic ‘grey digital divide’ in Hong Kong and had three objectives. First, it aimed to devise a typology using LPA to organize individual differences in motivation, access, digital skills, and usage of smart devices and online activities. Second, it explored the demographic, health, and help-seeking preferences associated with digital engagement profile membership among the younger and older participants. Third, it investigated the association between digital engagement profile membership and frequencies of different types of social contacts.

In summary, we found that the older group (aged 55 years or above) was still lagging their younger counterparts (aged 18–54 years) on most indicators of digital divide, from optimism (motivation level), range of devices owned (access level), skills on social media, information search, e-payment, document handling and troubleshooting (digital skills level), to the duration of device use and usage of a range of apps (instant messaging or social media, financial transactions, entertainment, daily life information), but not insecurity and the use of apps for health purposes. These differences were largely recovered when comparing participants aged 55–64 years to those aged 65 years or above. We then submitted these 16 indicators into LPA which resulted in three latent profiles, namely Proficient, Intermediate, and Novice. The older group was over-represented in Novice and Intermediate compared to their younger counterparts. In both age groups, gender, age, occupational status, education, and the number of relatives residing abroad differentiated the membership of digital engagement profiles. In the older group, the number of co-living family members and self-ratings of health further distinguished participants with different profile membership. Also, among both age groups, participants were most likely to consult their family about technological problems but not peers or social services, and Intermediate had the highest frequencies of help-seeking. After accounting for demographic factors and their interactions with age, profile membership was associated with various types of social contacts beyond those with family and relatives. As hypothesized, more digitally proficient profiles were linked to more social contacts compared to less proficient profiles.

### Capturing digital divide with a typology

Recent studies have begun using a typology approach for distinguishing pockets of digital populations with a variety of indicators. A study conducted in Slovenia differentiated their sample into four clusters (apprehensive, level-headed, savvy, and reluctant users) using four types of digital skills (operational, information-navigation, social, creative) and usage (cultural, economic, personal, social), and found these clusters being differentiated by a repertoire of demographic variables including age, education, occupation, types of settlement, income, etc [52]. Another study using a Belgian sample of older internet users revealed three groups – Basic users, Allrounders, and Selective users, based on nine internet activities (searching information, email, online banking, contact with (grand)children, social media, shopping etc) [53]. Again, they found gender, age, education, income and marital status significantly differentiated cluster membership. Liu et al [54] explored the changes in information and communication technology use before and after the pandemic among older Chinese and Korean Americans. They differentiated usage by not only types (e.g., use video calls, order groceries online, use email and texts), but also their frequencies before and during COVID, adding the temporal stability dimension to their clustering. They found three clusters – users with minimal online activities before and during the pandemic (Limited users), users with expanded online activities during the pandemic (Expanded users) and users with high levels of online activities before and during the pandemic (Active users). A recent study from Korea [55] identified three distinct profiles of digital literacy, namely, Low-level, Middle-level, and High-level, based on a sample of middle-aged and older adults. Their findings indicated that older adults with lower education levels, limited participation in in-person social activities, and a need for assistance with daily instrumental activities were more likely to fall into the Low-level category. Conversely, those with greater social support tended to be classified in the “high-level” group, which aligned with our findings. The use of LPA for identifying digital engagement profiles is fast-growing. Most resultant profiles of existing studies are differentiated by the amount (high vs low) and/or the breadth (extensive vs selective) of digital usage and skills, so as our findings. Our findings also show that some pairs of profiles were comparable on certain indicators while other indicators saw more differentiated levels among the profiles, providing support to the utility of using a typology approach. For instance, the extent of troubleshooting by oneself and the skill on using resource sharing apps were comparably low between Novice and Intermediate, whereas instant messenger and social media skill peaked in both Intermediate and Proficient. The uses of apps related to financial transactions and health purposes were both low in Novice and Intermediate. Besides, like previous studies, this study found that more advanced digital profiles are related to better socio-economic status and more social contacts. While these convergences lend support to the external validity of our findings, readers should be mindful that the choices of indicators, the sample characteristics, and the socio-cultural-technological landscape may significantly affect the resultant profiles and the generalizability of one typology to another context is not necessarily warranted.

Most extant studies still relied on just internet activities as indicators for profile generation [56,57] but seldom explored more than one level of digital divide, especially considering online activities and digital skills alongside motivational and access factors. As per the RAT [9], these factors form the first two levels of digital divide, providing an important impetus for predicting continued digital learning and usage. We also believe this is the first study in Hong Kong using a latent modelling technique to generate a theoretically informed typology of digital engagement, providing a consolidated view concerning the size and the form of digital divide, supplementing the findings from previous local studies [10–12,23]. We reckon this is a strength of the current study. Second, the high level of digitization, mandatory digital pandemic policy, accessibility for support and the government’s digital inclusivity initiatives in Hong Kong favors a narrow digital divide. Thus, our findings may provide a starting point for cross-cultural comparison with cities and economies with similar levels of digitization.

Motivational factors including optimism and insecurity have traditionally been construed as trait-like characteristics, but recent studies found that these factors are amenable to change by people’s technological experiences [58]. The review by Blut & Wang [58] further reported that the impact of these motivational factors on the perceptions of technology depends on the purpose of usage and country’s technological development, such that optimism is more positively related to favorable technological perception when the technology is for hedonic use and in more technologically advanced countries, whereas insecurity is more determinant of unfavorable technological attitudes for utilitarian technologies and in less technologically advanced countries. Our findings reveal that optimism was positively related to material access, literacy, and usage indicators, whereas insecurity was negatively related to only the use of financial transaction apps, literacy in resource sharing, and troubleshooting by oneself. The later three variables (use of financial transaction app, resource sharing and troubleshooting) could have been more contingent upon participants’ comfort level with technologies than other usage and literacy variables. Participants may have regarded these functions as ‘riskier’ or taxing their technological confidence. However, the perceptions of riskiness of digital functions are beyond the scope of this study and future studies may conceptualize what such ‘risk’ means for different digital populations. Nevertheless, our findings provide support to the RAT that motivational factors covary with the possession, literacy, and usage of ICT.

Besides, in the current study, the three latent profiles were significantly different on all indicators informed by RAT, except Insecurity. This suggests that while our participants were differentiated by their interest in technologies, digital device possession, digital skills, and extensiveness of usage, they appeared to agree on the indispensability and instrumentality of digital technologies in everyday life, which echoed with the early findings from Loges & Jung [14] regarding the centrality of the internet to daily lives. Interestingly, in contrast to what RAT predicts, those who were least optimistic about technologies were not participants with the least access, digital skills, and usage. It is possible that those who had some exposure to digital technologies would be more aware of their limitations than those who were least exposed. We therefore concur with Van Dijk [9] and other studies on digital disconnection [59,60] that more investigation is needed to contextualize and elaborate what exactly ‘wants-not’ are, especially among those who have had some experience using the technologies. This could be particularly insightful for understanding the behaviors of the small segment of Novice among the younger group, in other words, whether their low literacy and usage are a matter of choice or a result of experiencing qualitatively different barriers compared to their older counterparts.

This study referred to the Thematic Household Survey Report No. 77 [10] for deciding the age cut-off for the ‘younger’ and the ‘older’ samples for planned analyses. Still, over half of the older group belonged to the Proficient profile, which was populated by over 90% of the younger group. This suggests a considerable number of older adults have attained common digital skills on par with most of their younger counterparts. On the other hand, the reduction of the proportion of Novice to less than 1% among the younger group as opposed to 5.2% among the older group suggests we may have located the ‘boundary’ of the ‘grey digital divide’. In other words, our findings reveal a pattern of digital engagement that almost varnished among the younger population but remained apparent in the older population. This points to the harsh reality that a subset of older adults is still facing a multitude of unresolved challenges in using digital technologies [60].

### Nuances beneath the grey digital divide

RAT [9] offers a framework for understanding how personal and positional categories influence digital appropriation via moderating the access and usage of various resources, including support network and cultural capital. A gradated pattern with Proficient having better socioeconomic status (e.g., income, educational attainment, full-time employment) than Intermediate, and then Novice was reproduced in both the younger and the older groups. This corroborates previous findings that indicate a positive correlation between education [61–63 25], income [24,64,65] and full-time occupation [66] with use of the internet and digital skills potentially influenced by cultural capital and literacy. The effect of socio-economic status on digital divide remains strong regardless of age and even after the pandemic-related digitalization.

Regarding gender, in both the older and the younger groups, female participants featured with a higher proportion in Intermediate than Proficient and Novice. Studies have long documented a gender digital divide favoring men [67,68], with exclusion from technology education and design, limited free time for experimenting with new technologies, negative gendered stereotypes and social expectations, as well as financial or institutional constraints (e.g., being excluded from workforce or vocational training) as major barriers for women. However, the study by Friemel [69] with older adults in the Switzerland reported a varnishing gender divide on internet use after controlling education, income, technical interest, pre-retirement computer use, and marital status. Pantelaki et al [32] also remarked that while men are mainly Utilitarian users of the internet, women are heavily represented among Enjoyment users. Taipale et al [26] reported gender differences to be generally modest among traditional and online media, and in the case of reading e-books gender differences even varnished among the oldest age groups. The higher percentage of female participants in Intermediate than Novice could point to a potentially stronger support and online communicative network among women than men [70]. Thus, when it comes to the gendered pattern of digital usage among older adults, a nuanced view considering usage functions, as well as social capital is warranted.

Our findings found better self-rated health and having more co-living family members predicted membership in more advanced profiles in the older group, but not in the younger group. We also assumed that online means would be needed for maintaining relationships abroad and therefore having more immediate relatives abroad should correlate with more advanced profiles. Yet, Intermediate rather than Proficient consistently reported more immediate relatives abroad in both the younger and the older groups. Considering the recent wave of migration in Hong Kong, especially among middle-aged families with children [71], future studies may examine how the ‘grey digital divide’ may evolve with increasing need for cross-border intergenerational online communication.

Lastly, our findings show that despite the generally low frequencies of help-seeking for problems related to digital technologies, family remained as the primary source of support, compared to peers and social services. This echoes with previous studies suggesting that family is a natural source of support, especially for older people, when they meet technological difficulties [72,73]. However, the process of family members, particularly younger ones, teaching their older relatives is not without challenges, including those related to adjusting teaching strategies as per the abilities and needs of their older relatives and the relational tension arising from protection versus autonomy [74]. Filial piety could be a double-edged sword. On the one hand, filial piety may hinder older adults’ learning of technologies as they could rely on their younger relatives for online tasks (e.g., paying bills, connecting with overseas relatives). On the other hand, such values may lead their young relatives to perceive teaching their older relatives using technologies as an obligation. However, Tang et al noted that some older adults acknowledged that their adult children were impatient or struggling when teaching them, especially in the cases concerning complicated maneuvers. Learning about the struggles and impatience of their adult children, some older adults refrained from asking further questions from their adult children. Thus, digital inclusivity initiatives provided by social services are essential, yet currently underused as shown in our findings.

### Digital engagement and social contacts

Previous studies often report a positive association between digital engagement and social contacts [75,76], where older adults with higher self-efficacy in using digital devices are more likely to engage in digital communication with their families [77]. Yeung et al found that the well-being benefits of leisure and social use of ICT was mediated by enhanced social support among local older adults. However, our findings enrich the RAT framework by showing that different types of social contacts are differentially sensitive to digital engagement. Specifically, digital engagement profiles did not predict the frequency of contact with family/relatives, but only those with neighbors/ friends/ co-workers and various organizations. Among the younger group, compared to Proficient, Novice had fewer contacts with neighbors/ friends/ co-workers, whereas Intermediate had fewer contacts with government/ religious organization/ political parties. Among the older group, Novice had fewer contacts than Proficient with schools/ hospitals/ social service agencies. It appears that online activities affect contact with relatively ‘weak ties’ [78], which provide valuable resources (e.g., information) that participants’ inner circle of (co-living) relatives may not possess. Haythornthwaite [79] further put forward that new media may provide a platform for activating ‘latent ties’, that are those with uncommunicated but technically connected others, into ‘weak ties’, as well as substantiating ‘strong ties’ through multifaceted means. Yet, in the case of Hong Kong as families live in proximity communication could be principally physical or adequately sufficed by common instant messengers that saw a capped skill among most participants. On the other hand, social contacts may influence the social and cultural capital needed for appropriation and usage of technologies as RAT [9] postulated. This could be particularly realistically mediated by the family [72–74] and peer support [80] for technological usage and learning, especially among older adults. As our study was cross-sectional in nature, our findings could not delineate the direction of causality between social contacts and digital engagement. A more realistic picture could involve paths of both directions, with social contact enhancing digital engagement through social and cultural capital, as well as digital engagement providing additional conduits for social contacts.

### Strengths and limitations

Besides the convenience provided by LPA through generating a definable number of profiles for group comparisons, our study has the following strengths. First, the sample was recruited through random digit dialing, providing more credibility to its representativeness. Phone interviews allowed the inclusion of people who do not have (good) access to the internet, preventing the exclusion of digital non-users. Second, the validity of the 16 indicators were backed up by an elaborate theory of digital divide, namely the RAT [9] and were supported by local experts and a prior qualitative study with local older adults [44].

This study has several limitations. First, the primary goal of this research was to explore the digital divide between younger and older citizens of Hong Kong. Thus, we employed a purposive sample that ensured comparable sample sizes on the younger (aged 18–54 years) and the older (aged 55 years or above) groups, but without finer divisions beyond aged 65 years. Future studies that aim at typologizing the digital engagement of the general population may recruit a non-purposive sample with a wider range of age groups. Considering the differences in physical and cognitive capabilities as well as technological exposure between young-, mid- and old-old individuals, we call for future studies to utilize finer demarcations at older ages.

Second, despite our best effort to obtain expert and laymen validation for the indicators, our variables, which aim at providing a broad coverage of the four digital divide levels, are by no means exhaustive of the range of motivation, access, digital skills, and usage pertinent to the well-being of the local population. While the insecurity subscale of TRI 2.0 measures concern over the reliability of technologies and fear of not being able to use them effectively, other more specific psychological barriers like privacy concerns, anxiety about financial fraud, and ageism may lead older adults to deliberately avoid certain digital platforms. These deterrents are equally influential in shaping digital engagement, but the use of telephone survey restricted the number of items we could have included in this study. Also, the choice of indicators was delimited by the existing socio-technological landscape, such that digital divide may be better measured by other indicators in societies with different social, political, economic, and technological characteristics. Thus, we encourage future studies to either broaden the variety of indicators when focusing on typologizing users on a single digital divide level, say motivation, and use different indicators as per the prevalent digital ecosystem and socio-cultural norms to reflect how discrepant digital engagement affects well-being of their population.

Third, RAT does not theorize explicitly how specific socio-cultural norms on factors such as familial support and the changes in the digital ecosystems and digital inclusion policies impact on the impetuses and consequences of, as well as the digital divide itself. On an individual level, these factors may also cause a person to transit from one profile to another profile. People’s digital engagement may change as per their digital exposure and/or support. In fact, the probabilistic nature of ‘soft-clustering’ in LPA means a person have different probabilities of belonging to the resultant profiles. While the current analysis assumed assignment of profile to each participant based on the largest probability, it is possible that some individuals straddled across adjacent profiles with similarly high probabilities. A shift in one of the indicators, say motivation or usage, may change their profile membership. Acknowledging such fluidity, large-scale surveys, preferably using a longitudinal panel, should be conducted periodically to track the evolution of digital divide in a population, transition of profiles in a person, and their implications on different population segments. Unlike theories such as Technological Acceptance Model (TAM) [81] or Unified Theory of Acceptance and Use of Technology 2 (UTAUT2) [82], RAT does not focus on the attitudinal factors affecting the acceptance and usage of *particular* technologies. Qualitative studies will help provide contextualized information regarding how attitudes, socio-economic statuses, cultural norms, policies, and sources of support affect digital engagement. For instance, our study compared technological help-seeking from family, peers, and social services among different digital engagement profiles, and found that most people turned to their families for support when they encounter technological problems, while digital support from social services appeared to be underused. Our findings also reveal the association of digital engagement with the number of co-living relatives and number of relatives living abroad, which may point to the potential role of family-related incentives for using digital technologies for communication. However, our data is limited in testing and reflecting how family, policies, and other social factors dynamically shape digital engagement.

The current study is constrained by the reliance on self-reported items considering the need to obtain a large enough dataset within a short period of time to provide a representative snapshot of the digital engagement pattern of a population. These items may suffer from report biases due to social desirability or participants’ inadequate understanding of the functions featured in the items. For instance, individuals with limited technological exposure may not possess adequate understanding of the functions in the items and potentially over- or under-estimated their literacies and usage. Future studies may consider using behavioral measures, such as asking participants to complete certain digital tasks in a controlled setting for more accurate reflection of their skills or rely on available usage or meta-data from online service providers for capturing usage patterns. However, compared to survey data, the former may not accommodate a large enough sample size within a short while, and the latter may include only people with extensive online activities. Thus, either way may compromise the representativeness of the sample. Thus, future studies should consider the pros and cons of different methods of assessment and whether they commensurate with the research objectives.

Lastly, despite relying on random digit dialing to household and mobile phone numbers for sample recruitment to avoid excluding those were not ‘online’, all participants reported having at least one digital device for accessing the internet. The sampling and design of this study precluded a focused investigation on digital disconnection [59,60]. It is likely that those who are disconnected entirely from the digital ecosystem may either experience extensive barriers, including physical, cognitive or economic hardships, being institutionalized in care homes or incarcerated, or voluntarily withdraw for idiosyncratic reasons. For either of the above reasons, a separate study will be needed to explore their experience and challenges.

### Practical implications and future directions

According to the ITU [5], digital inclusivity encompasses providing reliable and affordable internet connectivity and devices, offering training and resources to develop necessary digital skills, ensuring that digital content is accessible and relevant to diverse audiences, including those with disabilities, and encouraging active participation in the digital economy and society. Thus, digital inclusivity covers equity concerning distribution, usage, and participation in ICT. Our findings may inform practical implications on multiple levels of digital divide, from enhancement of motivation, to enabling equitable access to digital infrastructure and devices, skills training, and incentivizing usage. The typology fosters diversification of digital support and streamlining of valuable resources to the most needed. While indicators with which Novice and Intermediate were equivalent (e.g., using apps for financial transactions and health records, troubleshooting by oneself) may screen out proficient users, indicators where Intermediate and Proficient saw no difference may suggest a ubiquitous phenomenon, with instant messagingand social media skill and usage being one of such, which could be capitalized for care service provision.

For Novice, even though they had the lowest skills and usage, they were not the most reserved about new technologies. These 5% of older adults possessed a pattern of digital engagement that almost varnished among their younger counterparts. These findings provide a strong rationale for community digital support to even those who are the most novice. Government and regulatory bodies may incentivize technology developers to engage older adults in designing or co-creating technologies and related services which may enhance their age-friendliness and relevance, such as in terms of the literacy, cognitive, and dexterity requirement of the interface, thereby reducing the barrier for access and usage [83,84]. Considering the strong association between socio-economic status and digital profile membership, subsidizing access to devices may be targeted for Novice with low household income, or who are not in full- or part-time workforce. These could be in the form of means-tested low-cost device rental, sharing of unused broadband networks, or cash subsidies for purchasing devices or broadband services. A program that tracks, reminds and incentivizes usage may follow from these device rental or subsidy schemes. Future studies are needed to gauge the effectiveness of such programs for enhancement of access and usage sustainability.

For Intermediate, the support focus could be on skills enhancement, as most of them had a device but remained highly selective in terms of their usage, focusing on mostly social media and instant messaging. They were also the group that were most likely to seek help. Enhancement of literacy may potentially reduce their apprehension to new functions and increase their scope of usage. The review by Schirmer et al [85] on 47 studies on promoting digital competence of older adults highlights the need for flexibility and addressing a relevant challenge for promoting digital skills. Their review emphasizes the value of participatory design for rendering the training curriculum relevant to the needs of the participants, social support, and maintaining a welcoming environment for learning. Hence, for Intermediate, as they had the lowest optimism with technologies but a fair exposure to the digital world as per their proficiency in instant messaging and social media related functions, digital learning may target reducing their concerns about using new technologies for instrumental functions, say financial or health management. However, skills learning could be prohibited by the lack of opportunities to use them beyond the ‘classroom’. Thus, for a skills training program to be effective, subsequent usage could be incentivized by a reward system, and beyond-program continuous support (e.g., clinics for individual trouble-shooting or booster sessions) should be made available. Furthermore, our findings show that digital engagement was more strongly related to the frequency of contact with ‘weak ties’ or even ‘latent ties’, which could be important information sources, especially for obtaining health and social care support for older adults. Kim & Feng [86] reported that compared to younger participants, older participants were less likely to reciprocate on social media and emails, regardless of both quantity and willingness of reciprocation, and that such age difference is mediated by the ability to use communicative technologies. Online social exchanges could be the conduit toward economic, political, cultural, and institutional participation. Thus, digital support may facilitate the use of digital media to access personally relevant information and services, especially among the Intermediate. While our study did not measure the usage of social and health care services, future studies may elaborate how digital users vary in terms of their access to and usage of these formal services, and whether such skills enhancement programs augment the usage of these formal services, especially among digital users characterized by an Intermediate profile.

For Proficient middle-aged and older adults, they may be invited to engage in co-creating digital solutions for their less tech-savvy counterparts by engaging in test-bedding of assistive technology and smart devices [83,84]. Tech companies should regard these adults as their target market segment, instead of seeing this large segment of consumers as ‘irrelevant’. Take telehealth for peri-and post-menopausal women as an example. To improve perimenopausal and postmenopausal women’s well-being, Menotech [87,88], as a branch of Femtech that encompasses primarily smartphone apps and telemedicine platforms aimed at managing symptoms, hormonal replacement therapy, and triaging low-risk women for regular check-ups, has flourished in recent years in North America, Europe, and Australia. Proficient middle-aged and older adults could be incentivized to use these cutting-edge technologies for managing their health, considering their current under-usage of health-related apps based on our data. Besides being the consumers of the latest technologies, Proficient middle-aged and older adults may be recruited for supporting their Novice or Intermediate counterparts in community digital inclusion initiatives as peer coaches. Ma et al [80] found that from a generational perspective, observing other older adults led to more effective learning of technologies and greater self-efficacy and willingness to use among older learners than observing younger or children models. It is hoped that by deploying more same-aged coaches, the usage of public digital support could be enhanced. The engagement of older digital users for test-bedding technological products or instructing their peers has been empirically explored overseas. Yet, their effectiveness to local proficient older users and the gerotechnological ecosystem at-large awaits empirical investigation.

While the assessment of whether the enhanced digital adoption by local older adults, potentially attributable to the pandemic, is sustainable, as well as the attrition of skills due to sporadic usage or changes in the interface and software settings are beyond the scope of this cross-sectional study, the sustainability of these digital habits could be informed by older adults’ attitudes to the technologies in question, including their perceived usefulness, perceived ease of use, support, hedonic value, as per models such as TAM [81] and UTAUT2 [82]. Accordingly, if older adults see their current digital habits fulfilling their needs for maintaining close bonds, making their lives convenient and fun, they could be more likely to sustain their digital engagement. This may be elaborated by a longitudinal study based on TAM, UTAUT2, or similar models targeting a particular technology. Conversely, digital fatigue, including physical, social, motivational, and emotional fatigue from excessive digital engagement, was evidenced among students and employees who have been virtual schooling or working for a sustained period, especially during the pandemic, adding to their stress and frustration [89,90]. While our data did not show a long period of daily digital engagement among our older adult participants, the prevalence, characteristics, and impact of digital fatigue on the well-being and long-term digital engagement of older adults remains largely unknown. Continuous support is vital for addressing these new challenges brought by the evolving socio-cultural-technological landscape and maintaining older adults’ motivation and digital usage. For instance, in addition to rounds of community programs for enhancing digital competence of older adults and intergenerational digital inclusivity, the Hong Kong government announced a budget of $100 million Hong Kong Dollars in 2024 for setting up community-based helpdesks across the territory for providing fixed-point training and troubleshooting for older adults, especially those living alone or in underprivileged areas. It is hoped that these helpdesks will provide individualized and sustainable support that can flexibly adapt to the changing technological needs of older adults. Certainly, the suggestions above, including subsidies for devices, incentivizing usage, and others could be adaptable to the prevalent technological environment, given the service providers are sensitive to the changing needs of their older users. This in turn highlights the importance of periodical investigation of the transforming digital divide as per the technological development to offer empirical data for service innovation.

There is an ongoing debate on a varnishing digital divide as digital technologies become potentially accessible to even the most deprived populations and ‘digital natives’ become the majority in societies [91]. As our findings still illustrate systemic inequalities in digital engagement by, say, age, socio-economic status, and gender across both the older and the younger groups in a highly digitized city like Hong Kong, these inequalities should be duly considered when designing different digital training and access programs. Implementing gender-sensitive programs or initiatives targeting low-income groups, caregivers, or the oldest-old individuals may increase the relevance of the content for the intended audience, thereby enhancing their adoption and effectiveness.

Going beyond individual-level impetuses, Hatuka and his team [92,93] proposed a framework that connects demographic attributes, neighborhood infrastructure and digital usage. The framework proposes that the ‘geography’, including the spatial, mobility, built-form, and amenities characteristics of a neighborhood, interacts with the demographics to influence individual and neighborhood digitization. Regarding infrastructural disparities, the household internet penetration has reached an impressive 98% [42] in Hong Kong. Additionally, there are over 85,000 Wi-Fi hotspots available for public use [95]. Hatuka’s framework may help to account for ‘neighborhood-based’ digital divide that exists outside the robust infrastructure, and touch upon accessibility to support system and availability of physical alternatives as factors leading to neighborhood differences in digital engagement. As the next step of our project, we will employ Geographical Information System to explicate the associations of digital engagement with neighborhood characteristics, to supplement the currently individualized perspective of digital divide with a more ecological view. These future findings may help streamline support resources more accurately as per the needs of different districts.

Pirhonen et al [96] compared the qualitative data from Finland, a highly digitized but fast-aging society, and Ireland, a relatively less digitized and younger society, and reached at the ‘Janus-faced digitization’ framework describing both optimism and drawbacks older adults perceive toward technologies. Some aspects that constrain older adults’ digital engagement could be related to biological ageing, such as deteriorating visual and hearing functions, finger dexterity issues due to arthritis, declining memory and cognition, etc. These barriers could potentially be rectified by inclusive designs, which refers to an approach to create accessible products and experience through understanding consumer diversity and enabling the product or service to respond to the needs of more people [97]. These could involve accessibility settings on a regular device, assistive tools, as well as smart devices and apps with tailored and simplified functions or interfaces, enabling people with sensory, physical, or mild cognitive limitations to enjoy the benefits of digital technologies. However, other drawbacks suggested by Pirhonen et al [96], including demands for constant learning the fast-changing technologies, deepening inequalities in access to support, and a loss of human touch in services were not adequately addressed in the current study but are important aspects to be mindful of when developing age-inclusive digital technologies and services. Consideration on these aspects leads us to close this section with a reflection on what if some older adults prefer non-digital interactions, or (the learning of) using digital technologies is too demanding that does not commensurate with the benefits they may gain. In fact, as of 2023, the smartphone penetration rate among older adults in Hong Kong reached a ‘bottleneck’ at 88.0% [94]. Despite the potential benefits of digital engagement to the well-being of older adults, it is important to respect individual preferences for disengagement from (chasing after) the ever-expanding online world. Thus, off-line choices should remain available to those who prefer or are unable to keep ‘online’, such that digital technologies would not become hegemony.

## Conclusion

This study aimed at generating a theory-informed typology to organize individual differences on motivation, access, digital skills, and usage, thereby showing the post-pandemic ‘grey digital divide’ in Hong Kong. Three latent profiles were revealed, namely Proficient, Intermediate, and Novice. Over 90% of participants aged between 18 and 54 years belonged to the Proficient profile, in contrast to less than 1% with a Novice profile. Among participants aged 55 years or above, the percentages of people belonging to the Proficient profile dropped to 59.2%, with 5.2% being Novice. Membership of these profiles was robustly associated with socio-economic status, and such a gradated relationship was reproduced in both the younger and the older groups. Additionally, membership of profiles was related to contacts with friends and various organizations, but not with families. This study advanced existing studies on digital divide as it consolidated statistically individual differences across different digital divide levels, from motivation to access, skills, and usage. Such an approach informs the development of community digital initiatives covering motivation enhancement, augmentation of material access, skills training, and usage reinforcement, in lieu of the characteristics of user groups in different digital engagement profiles.

## Supporting information

S1 Table Intercorrelations of the 16 indicators. (DOCX)
