# Supplementary material for: Revealing the nuances of ‘Grey Digital Divide’ in Hong Kong: A latent profile analysis
Source: PLoS One. 2025 Jul 9;20(7):e0326413. doi: 10.1371/journal.pone.0326413 (PMC12240337; doi:10.1371/journal.pone.0326413)
Supplement: S1 Table — (DOCX) [file pone.0326413.s001.docx]

**Supplementary table 1: Intercorrelations of the 16 indicators**

| **Digital divide levels** | **Indicators** | **1** | **2** | **3** | **4** | **5** | **6** | **7** | **8** | **9** | **10** | **11** | **12** | **13** | **14** | **15** | **16** |
| --- | --- | --- | --- | --- | --- | --- | --- | --- | --- | --- | --- | --- | --- | --- | --- | --- | --- |
| Motivation | 1. Optimism | - |  |  |  |  |  |  |  |  |  |  |  |  |  |  |  |
|  | 2. Insecurity | .012 | - |  |  |  |  |  |  |  |  |  |  |  |  |  |  |
| Material access | 3. Ownership of four types of digital devices | .160^**^ | -.000 | - |  |  |  |  |  |  |  |  |  |  |  |  |  |
| Digital skills | 4. Troubleshooting by oneself | .196^**^ | -.092^*^ | .449^**^ | - |  |  |  |  |  |  |  |  |  |  |  |  |
|  | 5. Information searching | .094^*^ | -.016 | .362^**^ | .388^**^ | - |  |  |  |  |  |  |  |  |  |  |  |
|  | 6. Using messaging or social media applications | .006 | -.008 | .201^**^ | .183^**^ | .384^**^ | - |  |  |  |  |  |  |  |  |  |  |
|  | 7. Document handling | .174^**^ | -.027 | .501^**^ | .541^**^ | .473^**^ | .280^**^ | - |  |  |  |  |  |  |  |  |  |
|  | 8. E-payment | .253^**^ | -.054 | .483^**^ | .544^**^ | .496^**^ | .313^**^ | .627^**^ | - |  |  |  |  |  |  |  |  |
|  | 9. Resource sharing | .172^**^ | -.109^*^ | .488^**^ | .591^**^ | .343^**^ | .174^**^ | .610^**^ | .544^**^ | - |  |  |  |  |  |  |  |
| Usage | 10. Messaging or social media | .191^**^ | .013 | .398^**^ | .396^**^ | .393^**^ | .357^**^ | .440^**^ | .484^**^ | .406^**^ | - |  |  |  |  |  |  |
|  | 11. Apps related to financial transactions | .311^**^ | -.108^**^ | .461^**^ | .536^**^ | .375^**^ | .217^**^ | .536^**^ | .651^**^ | .531^**^ | .503^**^ | - |  |  |  |  |  |
|  | 12. Apps related to health records | .125^**^ | .006 | .277^**^ | .157^**^ | .140^**^ | .104^*^ | .183^**^ | .211^**^ | .142^**^ | .207^**^ | .252^**^ | - |  |  |  |  |
|  | 13. Apps related to entertainment | .186^**^ | .018 | .293^**^ | .305^**^ | .319^**^ | .180^**^ | .290^**^ | .310^**^ | .320^**^ | .351^**^ | .352^**^ | .161^**^ | - |  |  |  |
|  | 14. Apps related to daily life information | .239^**^ | -.027 | .329^**^ | .354^**^ | .331^**^ | .186^**^ | .349^**^ | .396^**^ | .328^**^ | .440^**^ | .434^**^ | .274^**^ | .337^**^ | - |  |  |
|  | 15. Average hours spent on smartphones, daily | .116^**^ | .028 | .161^**^ | .243^**^ | .189^**^ | .059 | .176^**^ | .214^**^ | .231^**^ | .330^**^ | .276^**^ | .024 | .214^**^ | .136^**^ | - |  |
|  | 16. Average hours spent on laptops / computers, daily | .063 | -.057 | .392^**^ | .381^**^ | .259^**^ | .143^**^ | .408^**^ | .342^**^ | .425^**^ | .275^**^ | .325^**^ | .058 | .206^**^ | .207^**^ | .274^**^ | - |
|  | ^**^*p* < .001, ^*^ *p* < .05 | | | | | | | | | | | | | | | | |
